# Prior Engagement in Physical Activity Correlates with Enhanced Quality of Life Perceptions among Older Adults during COVID-19 Lockdown

**DOI:** 10.3390/brainsci14080765

**Published:** 2024-07-29

**Authors:** Gian Mario Migliaccio, Cesar Ivan Aviles Gonzales, Goce Kalcev, Elisa Cantone, Marcello Nonnis, Antonio Urban, Sonia Marchegiani, Samantha Pinna, Massimo Tusconi, Diego Primavera, Mauro Giovanni Carta

**Affiliations:** 1 Department of Human Sciences and Promotion of the Quality of Life, San Raffaele Rome Open University, 00166 Rome, Italy; gianmario.migliaccio@uniroma5.it; 2 Maxima Performa, Athlete Physiology, Psychology, and Nutrition Unit, 20126 Milano, Italy; 3 Nursing Program, Faculty of Health Sciences, Universidad Popular del Cesar, Valledupar 200002, Colombia; 4 Department of Medical Sciences and Public Health, University of Cagliari, Monserato Blocco I (CA), 09042 Cagliari, Italy; elisa.cantone@libero.it (E.C.);; 5 Department of Pedagogy, Psychology, Philosophy, University of Cagliari, 09123 Cagliari, Italy; marcello.nonnis@unica.it; 6 University Hospital of Cagliari, 09124 Cagliari, Italy; massimotusconi@yahoo.com; 7 Department of Mental Health, ASL Medio Campidano, 09020 Cagliari, Italy

**Keywords:** quality of life, SF-12, elderly people, physical exercise, psychology

## Abstract

Background: This longitudinal study aimed to evaluate whether prior engagement in a physical exercise program correlated with enhanced perceptions of quality-of-life components among older adults during the COVID-19 lockdown period. Methods: The cohort comprised elderly individuals (aged ≥ 65 years) who had previously partaken in a 12-week randomized controlled trial investigating the effects of a mixed aerobic–anaerobic, moderate-intensity exercise program. Participants’ health-related quality of life was assessed using the Short Form Health Survey-12 item (SF-12) at the beginning of the initial trial and, again, one year later during the COVID-19 lockdown. In the exercise group, 44 participants were included, while the control group consisted of 49 participants, with computer-based, double-blind randomization conducted in Cagliari, Italy. The differences in scores for each SF-12 item between the two groups from T0 to T1 were compared using one-way ANOVA with Bonferroni corrections. Data were analyzed using the Statistical Package for Social Sciences (SPSS) version 27. Results: No statistically significant differences were observed on average by age (exercise group vs. control group 72.20 ± 4.78 vs. 72.91 ± 4.77; F = 0.513, *p* = 0.476). A decrease from T0 to T1 towards a better score on the SF-12 was observed in the exercise group compared to the control group in item 1 (F = 67.463, *p* < 0.0001); in item 5 (F = 4.319, *p* = 0.041); item 8 (F = 4.269, *p* = 0.041); item 9 (F = 10.761, *p* = 0.001); item 10 (F = 170.433, *p* < 0.001); and item 11 (F = 4.075, *p* = 0.046). Conclusions: The results suggest that participation in a moderate physical exercise program one year prior may have equipped older adults with better coping mechanisms to navigate the stress and isolation imposed by the COVID-19 lockdown, as reflected by their enhanced scores on quality-of-life components pertaining to mental well-being. Exercise may confer a protective effect against the adverse psychological impacts of stressful events like the pandemic, even among older adults with chronic conditions. This study underscores the potential benefits of exercise interventions for promoting quality of life and preventing mood disorders in the elderly population.

## 1. Introduction

Research on Health Quality of life (H-QoL) dates back over 40 years [1,2,3]. H-QoL is a concept implying multidimensional aspects. This term not only indicates satisfaction with economic conditions or employment status as a measure of these kinds of indicators but also includes satisfaction with the environment of living and working, wellness perceptions of physical and mental health, satisfaction with recreation and leisure, optimism about one’s objectives in life, and satisfaction with friends [4,5,6]. The perception of health-related quality of life is a fundamental measure of the well-being of older people [7,8,9]. During this life period, chronic health problems are frequent, such as depressive and anxiety disorders, together with a decrease in cognitive performance; in other words, all aspects that can have a significant impact on the quality of life [10,11,12]. Vice versa, a low perception of quality of life is associated with a greater risk of depression and cognitive decline [13], but also with a worse course of chronic pathologies characteristic of old age [14,15,16,17,18,19]. It is well known that physical activity, together with other parameters, improves the quality of life of older adults [18,20].

In a global context where the prevalence of chronic and disabling diseases is rising with age, the implementation of interventions aimed at improving quality of life is extremely relevant to public health [20,21]. Studies indicate that engaging in consistent physical exercise for a minimum of three months improves the way adults and young adults with psychosocial disabilities perceive their quality of life [22,23]. Exercise has been considered one of the most important factors that can impact an older adult’s quality of life while living at home [24]. The number of people with disabilities and a lack of autonomy has increased as a result of the global population aging [25]. Moreover, a study showed that multidisciplinary approaches based on exercise combined with social leisure activities are essential to improving physical recovery and health-related quality of life after injury [23]. 

A 12-week randomized controlled trial (NCT03858114) on a mixed aerobic–anaerobic, moderate-intensity physical exercise program was conducted in elderly people (this way, elderly people with chronic diseases could also be included in the sample) [24]. The results of the trial, in addition to an improvement in cognitive functions, also highlighted an improvement in the quality of life and depressive symptoms [25].

The objective of the present study is to verify whether having conducted the physical training (one year before) may have had an impact on the perception of the quality of life one year later during lockdown in the people who had conducted the training (exercise group) in comparison to a control sample.

The cohort of elderly people analyzed in this study started a year earlier, at the start of the randomized-controlled trial (RCT). The last assessment was scheduled for April 2020 serendipitously, just as lockdown was instituted in Italy in March 2020 due to the COVID-19 pandemic. The stressful conditions of the rigid lockdown would add value to the possible improvement in quality of life if it were found. Since the lockdown was a period of potential risk for depressive and stress disorders [26,27,28,29], a possible elevation in the perception of H-QoL, as well as an indicator of a lower presence of depressive and stress disorders, can be considered an element of prevention, as a decrease in the perceived quality of life has been observed in sub-threshold spectrum conditions of mood disorders [30,31], and in conditions of stress and dysregulation of personal rhythms considered a vulnerability factor for mood and anxiety disorders [32,33].

## 2. Materials and Methods

### 2.1. Design and Study Sample

A cohort study (secondary analyses) was conducted on an old adult sample; previous studies involved a 12-week randomized controlled trial on mixed aerobic–anaerobic, moderate-intensity physical exercise (NCT03858114) in Cagliari, Italy [24] and were followed for one year. The sample was recruited through public notices, and the Italian Olympic Committee (CONI) contributed to recruitment through radio, TV, and newspaper advertising. People interested in participating were to contact the telephone number dedicated to their general practitioners. Each participant had to submit a medical certificate attesting to their suitability for non-competitive physical activity in order to be eligible for the trial.

The study included people aged ≥65 years, without a gender limitation, living at home, and with positive medical evaluation for non-competitive physical activity. People who were less than 65 years old, suffered from health conditions that made them unsuitable for this kind of physical activity, had a BMI (body mass index) > 35, or had the presence of psychosis or a brain disease were excluded. The sample was evaluated at the start of the RCT (T0) and 1 year later during the lockdown (T1). In the exercise group, 44 participants were included, while in the control group, 49 participants were included, using computer-based, double-blind randomization-generating permuted blocks and blind codes, masking both the participants’ identities and status and their activity [34].

### 2.2. Instruments

The self-report questionnaire SF-12 (Short Form Health Survey-12 item) [35,36] was adopted in the validated Italian version [37] as a measure of the H-QoL. The tool creates three distinct scores: total QoL, physical QoL, and psychological QoL. It provides this by measuring the various aspects of QoL, including social functioning, emotional status, pain, general health status, vitality, and mental well-being. These measurements are then regrouped into two sub-scales: physical and psychological quality of life. The total score (with a range of 12–47) resulted from the answers to each item using the Likert scale. Higher scores indicated a higher level of perceived H-QoL. The internal consistency was Cronbach’s α = 0.94. In this work, we examined the total score and the scores for the individual items of the scale.

### 2.3. Interventions

Exercise group activities: There were three weekly sessions for the exercise intervention: warm-up, active phase, and cool-down. A combination of anaerobic and aerobic exercises was included in the active phase. The range of physical activity was set between 40 and 59% of the heart rate reserve (HRR). Throughout the activity, the HRR was continuously tracked and sent to the professionals via a telemetry system.

Control group activities: The control group’s cultural and recreational activities were based on learning about the background of the local culture, going to historical locations, and receiving wellness education.

The amount of time spent in the exercise group and the control activities was the same.

### 2.4. Statistical Analysis

Data were input into Excel and analyzed using the Statistical Package for Social Sciences (SPSS) version 27. The comparison between means and standard deviations (numerical data) between the characteristics of the two study samples was conducted by means of ANOVA one-way statistics; the comparison between nominal data was performed by means of the Chi-square test. The comparison of the mean and standard deviation of the overall score of SF-12 within the two groups was carried out by means of an ANOVA one-way repeated-measures test. Using computer-based, double-blind randomization generators with permuted blocks and blind codes to mask the participants’ identities, status, and activities, 44 people were included in the exercise group and 49 participants in the control group. The comparison between the difference T0–T1 of the score of each SF-12 item in the two groups was conducted by means of ANOVA one-way statistics with Bonferroni corrections.

### 2.5. Ethical Aspect

The trial was conducted according to the Declaration of Helsinki and its revisions [38]. The study is registered on ClinicalTrials.gov (NCT03858114). The Committee for Medical and Health Research Ethics of the “Azienda Ospedaliera Universitaria di Cagliari” approved the study with the reference number PG/2018/15546, approved 25 October 2018. The researchers provided information about the study and informed the participants about the opportunity to discontinue their participation if they wanted. Written informed consent was requested and obtained from each participant before their involvement in the study.

## 3. Results

Table 1 shows the study sample characteristics comparing the two groups; no differences in statistical significance emerged in the distribution by gender or the average by age. As shown in Table 1, even chronic non-psychiatric diseases of mild-to-medium extents or those of a medium extent present a homogeneous frequency in the two groups compared. In both groups (the exercise group and the control group), a decrease in the total score of the SF-12 scale was observed from the first observation (before the RCT) to the second observation one year later (during the lockdown), but in neither group was the decrease (difference T0–T1) statistically significant.

Table 2 shows the changes from T0–T1 in each item of the SF-12 and the differences reached during the lockdown in the decreasing. It was observed that there was a decrease toward a better score in the exercise group compared to the control group in item 1: In general, would you say that your health is…” (F = 67.463, *p* < 0.0001); item 5: In the last 4 weeks, at work or in other daily activities, due to your physical health, have you had to limit certain types of work or other activities? (F = 4.319, *p* = 0.041); item 8: In the last 4 weeks, how much pain has hindered you in your usual activities (both at home and outside the home)? (F = 4.269, *p* = 0.041); item 9: How long in the last 4 weeks did you feel calm and serene? (F = 10.761, *p* = 0.001); item 10: How much time in the last 4 weeks did you feel full of energy? (F = 170.433, *p* < 0.001); and item 11: How much time in the last 4 weeks did you feel discouraged and sad? (F = 4.075, *p* = 0.046).

## 4. Discussion

The results of this work show that after a year of moderate physical activity, the sample of older adults presented a decrease in some items of the SF-12, or even an increase, which led to better scores compared to the control sample who had not carried out physical activity. It should be noted that the items in which better results are observed are related to general health, such as item 1: In general, would you say that your health is…”, or related to moods closely linked to stress and anxiety; item 9: How long in the last 4 weeks did you feel calm and serene?); item 10: How much time in the last 4 weeks did you feel full of energy?; or item 11: How much time in the last 4 weeks did you feel discouraged and sad? Less often or with less statistical force, they are linked to chronic pathologies (present in a large percentage of the sample), such as item 5: In the last 4 weeks, at work or in other daily activities, due to your physical health, have you had to limit certain types of work or other activities?; or item 8: In the last 4 weeks, how much pain has hindered you in your usual activities (both at home and outside the home)?

The concept of quality of life is a complex framework that is associated with various factors in the aging population, including autonomy, social networks, physical and cognitive functioning, and engaging in psychical activities. The link between H-QoL and disease occurrence with evident consequences has relevance in the elderly due to the high occurrence of chronic disabling disorders [39,40]. For example, in China, a high-accuracy model measured the years lived with disability (YLD) rate by applying three monitoring indicators from the Chinese national routine as predictor variables. This model provided a realistic and feasible solution for measuring health-adjusted life expectancy (HALE) at the national and, especially, regional levels [39]. In addition, global burden of disease (GBD) 2021 contributed with an updated, comprehensive set of the fatal burden of disease summarized with cause-specific mortality metrics and years-of-life-lost (YLLs) metrics for 288 causes by age and sex across 204 countries and territories from 1990 to 2021 [40].

In old adults, a good level of QoL can motivate salutary living and adherence to treatment and rehabilitation programs, contrasting negative outcomes and the loss of autonomy [41,42,43]. The availability of interventions to improve QoL is a relevant global health issue due to the world’s population of elderly increasing and, in parallel, increases in the burden of chronic and disabling diseases [44,45]. This trend is causing social and health costs to exponentially grow [46]. According to the World Health Organization (WHO), aging well needs to be a top priority globally. Even though some interventions will be universally useful, it will be crucial for nations to keep an eye on the well-being and productivity of their aging populations in order to recognize health trends and create programs that specifically address the needs that have been identified. In addition to providing all older adults with access to affordable healthcare, strategies that better prevent and manage chronic conditions must also take the physical and social environment into consideration. However, it is essential to be careful that these adjustments do not increase the gaps that contribute significantly to the poor health and functional limitations that we see in older age [45].

Previous research found that regular physical exercise for at least three months causes an improvement in the perceived quality of life in adults and young adults with mental health conditions [47], as well as in individuals with disabilities related to motor and neurological impairment [48]. Exercise has also been found to be effective at influencing the quality of life of older adults living at home [49,50,51]. A meta-analysis of randomized controlled trials (RCT) investigated the use of physical exercise at high or medium–high intensity, with sessions ranging from three to five times per week [52]. This approach can be useful in indicating the benefits of physical activity “in an ideal perspective”, but it is not practical for older adults living in the community who frequently have mild chronic conditions like diabetes and hypertension, which make high-intensity training unsuitable [53,54,55].

Regarding the lockdown, the COVID-19 pandemic had a significant effect on older adults’ quality of life proportionally to age [56,57,58,59]. This occurrence could be linked to biological and social vulnerability, which are often linked to social exclusion and isolation, in addition to previous medical history [60,61]. These circumstances were linked to the severity and mortality of COVID-19, as well as the context imposed by the health crisis, which worsened historical gaps between population groups [62,63]. As reported by the results of one study, elderly individuals who went outside less frequently were also more likely to report worsening health. Going outside encouraged physical activity, which contributed to better health in older people [58]. Recent studies have shown that quality of life, especially in old age, has been found to be predicted by a variety of factors. For example, regarding personality, a positive association was also found between quality of life and higher levels of openness to experience and agreeableness in personality dimensions [64]. Also, financial resources, health, and meaning in life directly and positively influence the quality of life [65,66].

The novelty of this study was, as highlighted in previous articles, that the data demonstrated that even medium-intensity mixed aerobic anaerobic exercise could have an impact on counteracting cognitive decline [24], improving depressive symptoms, and even the perception of H-QoL [25]. These new data highlight that one year after the start of the trial and nine months after the end, the group that conducted the exercise program showed a smaller impact of stress subsequent to the lockdown and a better level of certain components of H-Qol, particularly those linked to energy, general well-being, the state of calm, and the absence of negative thoughts. The practical implications of the study are similar to the results found from numerous studies on physical exercise and other rehabilitation interventions [67,68], which have shown that QoL is an outcome that improves more slowly than others. Therefore, considering the good adherence to the trial in our experience and the fact that moderate exercise does not excessively stress participants, we suggest conducting longer-term trials.

It should also be highlighted that energy, a sense of general well-being, and positive thoughts are excellent measures against the risk of depression and stress symptoms [69,70], which are so frequent during the lockdown [25,26,71]. It is, in fact, known that persistent negative thinking, pessimism, and rumination have a relevant role in the onset and relapse of depressive disorders in older adults. It is, therefore, positive that a usable physical exercise intervention can be an element of improvement in quality of life and prevention, even for adults who, like many people of this age, suffer from chronic disorders. Firstly, the chronic diseases of the elderly are a vulnerability factor for the risk of depression and the worsening of the quality of life [69]. Secondly, this type of intervention could have an impact on those sub-threshold forms of mood disorders, which are perhaps positive only at screening tests [72,73] and risk evolving into frank disorders when they experience conditions of stress [33].

Certain limitations need to be considered. While the sample size was relatively large compared to similar studies, it was insufficient to permit a multivariate analysis of the outcome determinants. Nevertheless, the sample was well-balanced with respect to the primary known confounding factors, including age, gender, and educational level. Additionally, the follow-up evaluation coincided with the lockdown, likely affecting our results. Despite this, the analysis revealed some very interesting findings, especially considering the stress individuals were subjected to during those phases of the COVID-19 pandemic and related lockdown.

## 5. Conclusions

The findings suggest that participating in a moderate physical exercise program one year before may have helped older adults better cope with the stress and isolation of the COVID-19 lockdown, as reflected in their higher scores on quality-of-life components related to mental well-being. Exercise may have a protective effect against the negative psychological impacts of stressful events like the pandemic, even in older adults with chronic conditions. The study highlights the potential benefits of exercise interventions for improving quality of life and preventing mood disorders in the elderly population.

## Figures and Tables

**Table 1:** Study sample characteristics and differences during the observation times in the exercise group and control group concerning the SF-12 total score.

	Exercise Group	Control Group	
	44	49	
**Female**	22/44 50%	28/49 57.1%	Chi square = 0.476; 1df *p* = 0.490
**Age**	72.20 ± 4.78	72.91 ± 4.77	ANOVA 1.91 df F = 0.513, df 1.92, *p* = 0.476
**With chronic non-psychiatric disease (mild)**	24 [54.5%] (13) [29.5%]	29 [59.1%] (16) [32.6%]	Chi square = 0.203; 1df *p* = 0.652 (Chi square = 0.104; 1df *p* = 0.704)
**SF-12 Tot T0**	35.86 ± 5.08	35.61 ± 3.95	ANOVA for repetitive measure F = 3.409, (1.87 df), *p* = 0.068
**SF12 Tot end**	34.22 ± 8.01	33.83 ± 6.04	ANOVA for repetitive measure F = 2.981, (1.97 df), *p* = 0.082

**Table 2:** Changes from T0–T1 for each item of SF-12 and the differences (exercise vs. control group) reached during the lockdown.

Item SF-12	Exercise (T0)		After 12 Months (T1)	Difference	F (df 1.92)	*p*
1. In general, would you say your health is…	Exercise (N = 44)	3.0 ± 0.73	3.23 ± 0.70	0.23 ± 0.08	67.463	<0.0001
	Control (N = 49)	3.14 ± 0.60	3.06 ± 0.62	−0.08 ± 0.23		
2. Your health currently limits you from carrying out activities of moderate physical effort such as moving a table, using the vacuum cleaner, playing bowls or going for a bike ride	Exercise N = 44	1.77 ± 0.42	1.79 ± 0.40	0.02 ± 0.15	3.403	0.068
	Control (N = 49)	1.65 ± 0.47	1.79 ± 0.40	0.13 ± 0.25		
3. Your health currently limits you from carrying out activities of moderate physical effort such as climbing a few flights of stairs	Exercise (N = 44)	1.77 ± 0.40	1.79 ± 0.49	0.02 ± 0.20	2.994	0.073
	Control (N = 49)	1.67 ± 0.47	1.76 ± 0.42	0.09 ± 0.19		
4. In the last 4 weeks, have you experienced at work or in other daily activities, because of your physical health, did you perform less than you would have liked?	Exercise (N = 44)	1.88 ± 0.31	1.83 ± 0.36	−0.05 ± 0.12	2.426	0.123
	Control (N = 49)	1.75 ± 0.43	1.75 ± 0.43	0.0 ± 0.16		
5. In the last 4 weeks, have you experienced at work or in other daily activities, because of your physical health, have you had to limit certain types of work or other activities?	Exercise (N = 44)	1.81 ± 0.38	1.80 ± 0.39	−0.01 ± 0.18	4.319	0.041
	Control (N = 49)	1.79 ± 0.40	1.70 ± 0.45	−0.09 ± 0.19		
6. In the last 4 weeks, because of your emotional state (such as feeling depressed or anxious), did you performed less than he would have liked?	Exercise (N = 44)	1.79 ± 0.40	1.86 ± 0.34	0.07 ± 0.19	2.977	0.88
	Control (N = 49)	1.75 ± 0.43	1.75 ± 0.43	0.0 ± 0.20		
7. In the last 4 weeks, because of your emotional state (such as feeling depressed or anxious), have you had a loss of concentration at work or other activities?	Exercise (N = 44)	1.81 ± 0.38	1.83 ± 0.42	0.02 ± 0.12	0	1
	Control (N = 49)	1.77 ± 0.41	1.79 ± 0.42	0.02 ± 0.19		
8. In the last 4 weeks, how much pain has hindered you in your usual activities (both at home and away from home)?	Exercise (N = 44)	4.36 ± 0.37	3.95 ± 0.89	−0.41 ± 0.17	4.269	0.041
	Control (N = 49)	4.28 ± 0.78	3.79 ± 0.91	−049 ± 0.20		
9. How long in the last 4 weeks did you feel calm and peaceful?	Exercise (N = 44)	4.47 ± 1.07	4.58 ± 0.92	0.11 ± 0.18	10.761	0.001
	Control (N = 49)	4.57 ± 0.98	4.55 ± 0.91	−0.02 ± 0.20		
10. How much of the time in the last 4 weeks did you feel full of energy?	Exercise (N = 44)	4.15 ± 1.31	4.37 ± 1.0.6	0.22 ± 0.17	170,433	<0.0001
	Control (N = 49)	4.32 ± 0.93	4.02 ± 1.10	−30 ± 0.14		
11. How much of the time in the last 4 weeks have you felt discouraged and sad?	Exercise (N = 44)	4.88 ± 1.04	5.00 ± 1.10	0.12 ± 0.18	4.075	0.046
	Control (N = 49)	4.79 ± 0.78	4.82 ± 0.80	0.03 ± 0.20		
12. In the last 4 weeks, how long has your physical health or emotional state interfered in your social activities, family, friends?	Exercise (N = 44)	4.15 ± 0.70	4.20 ± 0.73	0.05 ± 0.18	3.307	0.082
	Control (N = 49)	4.02 ± 0.74	4.00 ± 0.81	−0.02 ± 0.19		

## Data Availability

The datasets of this study will be not publicly available due to individual privacy rules.
